# Evaluation of Resting Serum Bile Acid Concentrations in Dogs with Sepsis

**DOI:** 10.3390/vetsci9110627

**Published:** 2022-11-11

**Authors:** Lara Baptista, Danica Pollard, Andrea Di Bella

**Affiliations:** 1 Paragon Veterinary Referrals, Red Hall Cres, Wakefield WF1 2DF, UK; 2 Independent Researcher, The Rodhams, Christchurch PE14 9NU, UK; 3 Southern Counties Veterinary Specialists, Forest Corner Farm, Ringwood BH24 3JW, UK

**Keywords:** liver, sepsis, cholestasis, inflammation, bile acids

## Abstract

**Simple Summary:**

There is paucity of information in the veterinary literature regarding bile acid concentrations in septic dogs. Nonetheless, in critically ill human patients, bile acids concentrations are suggested to be an early predictor of short-term survival. This study, therefore, aimed to assess resting bile acid concentrations in a population of septic dogs and ascertain if comparably to human medicine, septic dogs would have higher bile acid concentrations when compared to non-septic dogs. Medical records of dogs presenting to a single referral center over a twelve-year period were retrospectively reviewed. Twenty-six client-owned dogs with a diagnosis of sepsis were included. Two control groups of non-septic dogs (ill control and orthopedic control) were also included. Resting serum bile acid concentrations were significantly higher in the septic group compared to the non-septic group combined. However, no difference in resting serum bile acid concentrations was identified between the septic and the orthopedic control group alone.

**Abstract:**

Recent studies in the human literature suggest that serum bile acid concentrations could be an early predictor of short-term survival in critically ill patients. However, there is no available information in the veterinary literature regarding serum bile acid concentrations in dogs with sepsis. We aimed to evaluate if resting serum bile acid concentrations differ between septic and non-septic dogs. This was a retrospective observational study, of medical records at a single referral center over a twelve-year period. Twenty-six client-owned dogs diagnosed with sepsis were identified. Twenty-one dogs presenting with a non-hepatobiliary systemic disease and twenty-nine dogs admitted for an elective orthopedic procedure, considered otherwise healthy, were selected as control groups. Resting serum bile acid concentrations were significantly higher in the septic compared to the non-septic groups (ill control and orthopedic control groups). However, when assessing bile acid concentrations between groups individually, no difference was identified between the septic and the orthopedic control group. These results should be interpreted cautiously.

## 1. Introduction

Sepsis is defined as a life-threatening organ dysfunction, caused by a dysregulated host response to infection [1] and is associated with a high morbidity and mortality in both human and veterinary medicine [2,3,4]. In the veterinary literature, mortality rates associated with sepsis in dogs are reported to range from 21–68% [4]. 

The liver has a fundamental role in the systemic response to sepsis [5], and bacterial infections can lead to cholestasis without direct invasion of the liver by a pathogen [6]. The inflammatory mediators released during the infectious process activate pro-inflammatory cytokines, which are potent inhibitors of hepatobiliary transporter gene expression, which will impair transport of bile, resulting in hyperbilirubinaemia and cholestasis [2,6,7,8,9,10]. 

Sepsis-induced cholestasis is mostly related to Gram-negative bacterial infections [9] but can also be associated with Gram-positive cocci and bacilli [6]. Lipopolysaccharide is a potent initiator of local and systemic inflammation as is released into circulation and mainly cleared by the liver [9]. Kupffer cells respond by producing increased levels of cytokines such as tumor necrosis factor α (TNFα), interleukin-1b (IL-1b) and interleukin-6 (IL-6) [9,11]. The induction of these proinflammatory mediators, will reduce expression of bile acid transporters, such as the sodium taurocholate cotransporting polypeptide (NTCP) and bile salt export pump (BSEP) located at the basolateral and canalicular membrane, respectively, resulting in impaired bile secretion [11,12]. Furthermore, inflammation will also result in reduction of the expression of the multidrug resistance-associated protein 2 (MRP2) which is expressed on the canalicular membrane of the hepatocytes and has an important role on the biliary secretion of conjugated bilirubin as well as organic anions [12]. This cholestatic inflammatory response of the liver is not restricted to the hepatocytes [11] and at the bile duct, the proinflammatory cytokines will also reduce ductal bile secretion and alter cholangiocyte tight junction function [9]. The consequent accumulation of bile constituents, mostly bile acids, may induce cytotoxicity, further impairing hepatic bile secretion [13]. 

Cholestasis is reported to be a very early event in the sepsis process, and in human medicine, hyperbilirubinemia has been considered a poor prognostic indicator for septic patients [14]. Nonetheless, recent studies have concluded that serum bilirubin may not be a reliable marker of cholestasis in critically ill patients, as is unclear whether it is truly indicative of severe hepatobiliary dysfunction or merely reflects a transient and adaptative response during critical illness [10,15,16]. Conversely, serum bile acids concentrations (SBAC) have been reported to be an early predictor of short-term survival in a recent study that assessed the clinical role of circulating bile acids in a mixed cohort of critically ill human patients [10]. In the same study, SBAC and bilirubin concentrations were not only increased but also higher in critically ill patients that died within the first 28 days, compared to control groups. Nevertheless, SBAC showed better predictive properties than bilirubin, with the highest concentrations found in patients with septic shock, suggesting a possible role as a novel marker [10] and as a highly sensitive and specific biomarker in predicting mortality in septic patients [17]. Another study evaluating critical illness and circulating bile acids assessed postmortem liver biopsies in conjunction with pre-agonal serum analysis, and concluded that serum bile acid concentrations were much more increased during critical illness than the bilirubin concentrations [15]. 

In veterinary medicine, studies have been conducted to investigate the usefulness of bilirubin, albumin, cholesterol, serum amyloid A, C-reactive protein, serum C-type natriuretic peptide, protein C and antithrombin concentrations as prognostic biomarkers for sepsis [4,18,19,20,21,22,23,24]. However, there is paucity of data regarding assessment of serum bile acids concentrations in septic patients. Decreased biliary excretion of bile acids caused by functional cholestasis has been described in veterinary medicine as a consequence of sepsis in one dog, in a study that assessed cholestasis associated with extrahepatic bacterial infection in five dogs [18].

Considering the paucity of data, the aims of the present study were to assess the value of resting SBAC in a population of dogs diagnosed with sepsis. It was hypothesized that dogs with sepsis would have higher resting serum bile acid concentrations, likely due to sepsis-induced cholestasis, when compared to a group of non-septic dogs (ill and apparently healthy dogs) admitted during the same period. 

## 2. Materials and Methods

### 2.1. Ethics Approval

This study was reviewed and approved by the Royal College of Veterinary Surgeons Ethics Review Panel (reference 2021-39).

### 2.2. Case Selection

This was a retrospective, single-center, observational study. The medical records of all dogs that presented between 2008 and 2020 that were diagnosed with sepsis without evidence of hepatobiliary and pancreatic disease were reviewed. The diagnosis was based on hematology, biochemistry and diagnostic imaging findings.

Medical records were researched with the words “sepsis”, “septic shock” and “septic peritonitis”. To be included in the septic group, other than displaying evidence of an infectious etiology, documented on diagnostic imaging findings (which included radiography, ultrasonography, and computer tomography), echocardiography and/or cytology and culture of fluid samples, dogs in the septic group had to fulfill criteria of systemic inflammatory response syndrome (SIRS). Proposed criteria for the diagnosis of SIRS in dogs, consists of documenting two or more of the following four clinical signs: hypothermia (<38.1 °C) or hyperthermia (>39.2 °C); leukocytosis (>16 × 10^3^/μL) or leukopenia (<6 × 10^3^/μL), percentage of band neutrophils higher than 3% of the total leukocyte count; tachycardia (heart rate > 120 beats per minute; [bpm]) and tachypnea (>20 respirations per minute [rpm]) [25]. Dogs for which an infectious etiology could not be identified, with missing or incomplete records or that did not meet two or more of the criteria for SIRS, were excluded from analysis. Moreover, dogs diagnosed with sepsis that had a suspected or documented hepatic, biliary tract, or pancreatic disease, based on biochemistry results and diagnostic imaging findings, were excluded, as these conditions can alter resting serum bile acid concentrations. Dogs euthanized without treatment were also excluded. 

Medical records were also retrospectively reviewed to identify two control groups with complete records that were admitted during the same timeframe as the septic dogs. The first group included ill, non-septic dogs, presenting with a non-hepatobiliary or pancreatic systemic disease, to avoid intrahepatic/extrahepatic cholestasis, which could have interfered with the measurement of resting SBAC (ill control group). The second group included dogs that were admitted for an elective orthopedic procedure, deemed to be otherwise healthy, based on clinical history, physical examination findings, hematology, and biochemistry results (orthopedic control group). 

Data including the signalment, history, body weight (kg), heart rate (bpm), temperature (°C), serum biochemical parameters (albumin concentration [g/L], alanine-aminotransferase activity [ALT; U/L], alkaline phosphate activity [ALP; U/L], glucose concentration [mg/dL], total bilirubin concentration [mg/dL] and cholesterol concentration [mg/dL]) as well as hematology parameters (neutrophil count [×10^3^/μL]) were recorded. 

Measurement of resting SBAC [µmol/L] on admission, prior to administration of intravenous fluid therapy or antimicrobial therapy at the referral center, was necessary for inclusion. This parameter is included as a part of the routine biochemistry analysis offered by the external laboratory at the referral center where the study was conducted.

For dogs in the septic group, survival to discharge or whether they were euthanized/died was also recorded. 

### 2.3. Bile Acid Measurement

Resting SBAC were measured, as part of the laboratory investigations performed at the time of presentation, by the same external laboratory for all cases included in the study, by enzymatic cyclic colorimetric technique using a blood chemistry analyzer (Beckman-Coulter; model AU480, Brea, CA, USA) and Sentinel (Sentinel Diagnostics, Milan, Italy) was the reagent used for determination of resting SBAC. Daily quality control assessments are performed at the laboratory. The reference interval for resting SBAC considered by the laboratory is 0.1 µmol/L to 10 µmol/L, adopted from the literature, with subsequent verifications performed across several measurands at the laboratory. 

### 2.4. Statistical Analysis

Data were recorded in an electronic spreadsheet (Microsoft Excel, version 2010; Microsoft Corporation, Redmond, WA, USA) and were analyzed with a commercial statistical software (STATA: IC version 13; StataCorp. LLC. 2017. Stata Statistical Software: Release 15. College Station, TX, USA). 

Data were evaluated for normality of distribution using the Shapiro–Wilk test, in combination with visual assessment of histograms, with overlaid kernel density plots, and expressed as a mean and standard deviation (SD) or median with interquartile range (IQR) and range (minimum to maximum) depending on distribution. 

The relationship between sepsis and resting SBAC was assessed using the Mann–Whitney *U* test by comparing resting SBAC in septic and non-septic groups (ill and the orthopedic control groups combined). Further assessment of the relationship between sepsis and resting SBAC in each individual group (septic, ill and orthopedic control groups) was assessed using a Kruskal–Wallis test, with a further post hoc Dunn’s test with Sidák adjustment to assess pairwise differences between groups. 

The relationship between resting SBAC and other variables of interest recorded (including age, sex, body weight and biochemical parameters) was assessed using the Spearman rank correlation coefficient. A Mann–Whitney *U* test assessed if there was a relationship between resting SBAC and sex (female or male). 

The relationship between resting SBAC and other variables of interest in each individual group (septic, ill and orthopedic control groups), including signalment, biochemical and hematological parameters, was assessed to identify any potentially confounding associations and to allow for evaluation against biological parameters published in similar populations. The relationship between groups and non-normally distributed variables (albumin, ALP, ALT, total bilirubin, cholesterol and neutrophil count) was assessed using a Kruskal–Wallis test, with a further post hoc Dunn’s test with Sidák adjustment. The relationship between groups and normally distributed variables (glucose, body temperature and heart rate) was assessed using analysis of variance (one-way ANOVA), with a further post hoc Tukey’s test with adjustment for multiple comparisons, to identify pairwise differences between groups. For normally distributed variables, mean differences (including 95% confidence intervals [95% CI]) across groups are also presented. 

A Fisher’s exact test was used to assess the relationship between sex and group. 

For all statistical analyses, a *p*-value less than 0.05 was considered significant. 

## 3. Results

### 3.1. Groups Characterizations

Review of medical records identified 35 cases of septic dogs with nine cases being excluded due to incomplete records and/or evidence of a hepatobiliary disease. Therefore, a total of 26 dogs with sepsis were included in the study. There were 21 and 29 cases included in the ill and the orthopedic control groups, respectively. The median age of all dogs enrolled in the study was 94 months (IQR 64–116 months; range 3–163 months) and mean body weight was 22.8 kg (SD 12.1 kg; range 2.8–53.7 kg). There were several breeds represented, with the most common being Labrador retrievers (n = 17), English springer spaniels (n = 6), Staffordshire bull terriers (n = 4) and Cocker spaniels (n = 4). There were 31 females (40.8%; 29 neutered [93.5%] and 2 entire [6.4%]) and 45 males (59.2%; 30 neutered [66.7%] and 15 entire [33.3%]), with male dogs representing 81%, 61.5% and 41.4% of dogs included in the ill control group, septic group and orthopedic control group, respectively. 

### 3.2. Non-Septic Control Groups (Ill and Orthopedic Control Groups)

Medical conditions for dogs in the ill control group included gastrointestinal disease in 11 dogs (52.4%) with two dogs presenting with chronic and nine dogs presenting with an acute gastroenteritis; chronic bronchitis in two dogs (9.5%), bronchial collapse, chronic rhinitis, sino-nasal aspergillosis, a nasal cyst, angiostrongylosis, chronic kidney disease, benign prostatic hyperplasia and urolithiasis in one dog each (4.8%). None of the dogs in this group required hospitalization in the intensive care unit or underwent surgery. All dogs included in the orthopedic group underwent tibial plateau level osteotomy surgery (TPLO) for treatment of cranial cruciate ligament rupture. 

### 3.3. Septic Group

Within the septic group, the source of sepsis was gastrointestinal in 17 dogs (65.4%); aspiration pneumonia in three dogs (11.5%), endocarditis in two dogs (7.7%), pyothorax and aspiration pneumonia in one dog (3.8%), pyothorax only in two dogs (7.7%) and a lymph node abscess in one dog (3.8%). From the 26 dogs in the septic group, 11 (42.3%) underwent exploratory laparotomy. Abdominocentesis was performed in 12 (46.1%) of the 26 dogs and thoracocentesis in three (11.5%). Cytology of peritoneal effusion was performed in all 12 cases in which abdominocentesis was performed and culture in nine (75%) cases. Cytology of the peritoneal effusion was consistent with an exudate in all 12 cases with bacteria being identified in seven (58%) cases, and culture returned a negative result in four of the nine samples (44%). Of the positive culture results, three samples (60%) were positive for *E. coli*, one (20%) was positive for *E. coli* and *Enterococcus* and one (20%) was positive for *Staphylococcus* coagulase positive. Cytology and culture of the pleural effusion was requested in all three cases in which thoracocentesis was performed. Cytology was consistent with an exudate in all three cases and culture returned a positive result for *Staphylococcus* coagulase positive, in one case. Blood cultures were performed in the two dogs with endocarditis, and both samples returned a negative result. 

The median hospitalization period for dogs with sepsis was three days (IQR 2–6 days; range 1–19 days) and 46.2% (n = 12) of dogs survived to discharge. Of the 14 dogs that did not survive to discharge, nine (64.2%) were euthanised and five (35.8%) died in hospital, despite treatment. 

The results of comparisons across groups are summarized in Table 1. 

### 3.4. Resting Serum Bile Acid Results

When assessing both control groups together (ill and orthopedic groups), resting SBAC were significantly higher in dogs with sepsis (median 17.5 µmol/L; IQR 7.5–26.9 µmol/L; range 3–75.2 µmol/L) compared to this non-septic population (median 10 µmol/L; IQR 6.4–13 µmol/L; range 2–47 µmol/L; *p* = 0.023). 

**Figure 1:**
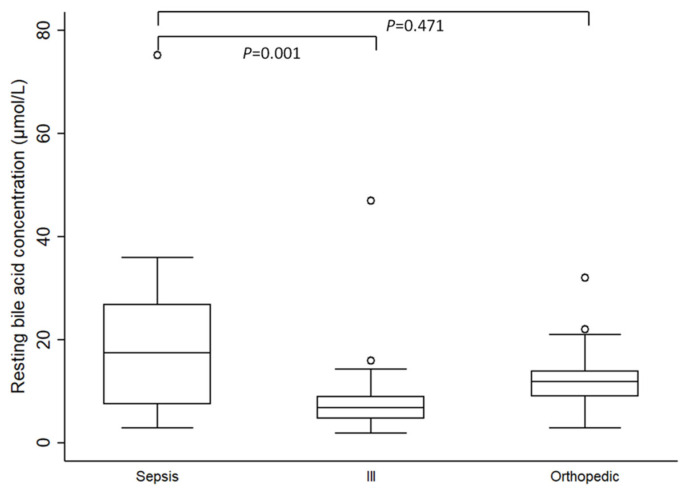
Comparison of resting serum bile acid concentrations in septic dogs (n = 26) with the ill (n = 21) and the orthopedic (n = 29) control groups. Boxes represent medians and interquartile ranges, and whiskers represent the range and individual points outliers. Septic dogs had significantly higher resting serum bile acid concentrations compared to ill dogs (*p* = 0.001) however there were no differences between the septic and the orthopedic group (*p* = 0.471).

Individual evaluation of resting SBAC between the septic group and both control groups revealed that resting SBAC were significantly higher in septic dogs compared to the ill control group (median 6.9 µmol/L; IQR 4.7–9 µmol/L; range 2–47 µmol/L; *p* = 0.001). However, there were no significant differences in resting SBAC between the septic and the orthopedic control group (median 12 µmol/L; IQR 9–14 µmol/L; range 3–32 µmol/L; *p* = 0.471) (Figure 1).

When considering the association between resting SBAC and other variables, a positive correlation with bilirubin was identified in the septic group (Spearman’s rho 0.59; *p* = 0.001). For the ill control group there was a negative correlation between bilirubin and resting SBAC (Spearman’s rho −0.43; *p* = 0.049) and there was no significant correlation between these two variables identified in the orthopedic group (Spearman’s rho 0.05; *p* = 0.789). 

No correlation was identified between resting SBAC and age (Spearman’s rho 0.10; *p* = 0.383) nor body weight (*p* = 0.585). Nevertheless, when considering sex, significant differences were found in resting SBAC, with female dogs displaying higher median resting serum bile acid concentrations (median 13 µmol/L; IQR 9–22 µmol/L; range 3–75.2 µmol/L) when compared to male dogs (median 9 µmol/L; IQR 5.5–15.7 µmol/L; range 2–47 µmol/L; *p* = 0.036).

When associating resting SBAC with all the other biochemical variables considered, only a positive correlation was found between resting SBAC and ALP (Spearman’s rho 0.27; *p* = 0.017). 

### 3.5. Other Results

When assessing the signalment, dogs in the orthopedic group had a higher median age (105 months; IQR 91–115 months; range 66–156 months) compared to dogs in the ill control group (69 months; IQR 47–102 months; range 5–148 months; *p* = 0.016). However, there were no statistically significant differences in age between the septic group and both non-septic groups (ill [*p* = 0.626] and orthopedic controls [*p* = 0.055]) nor in the body weight between septic and the ill (*p* = 0.076) nor the septic and the orthopedic control groups (*p* = 0.605).

Regarding the sex, there was a significant difference in distribution between groups with the orthopedic group having the highest proportion of female dogs (*p* = 0.021). 

Regarding the biochemical parameters, there were no significant differences in ALT (*p* = 0.435) and cholesterol (*p* = 0.442) between groups.

Albumin was significantly lower in the septic group compared to the ill (*p* < 0.001) and orthopedic groups (*p* < 0.001). When assessing ALP, it was significantly higher in the septic group, compared to the other two groups (*p* < 0.001). 

When considering both control groups together (ill and orthopedic groups) serum bilirubin concentrations were significantly higher in the septic group (median 4.5 mg/dL; IQR 3–15 mg/dL; range 1–48 mg/dL) compared to the non-septic dogs (median 2 mg/dL; IQR 2–3 mg/dL; range 0–5 mg/dL; *p* < 0.001). Furthermore, when evaluating this variable for each individual group, serum bilirubin was significantly higher in the septic group compared to both the ill (*p* = 0.01) and orthopedic control groups (*p* < 0.001). 

Glucose concentration on admission was significantly lower (−0.8 mg/dL; 95% CI −1.3–0.3 mg/dL) in the septic group compared to the orthopedic group (*p* = 0.001). When assessing hematological parameters, the neutrophil count was also significantly higher in the septic group compared to the orthopedic group (*p* = 0.007).

Heart rate on admission was significantly higher in the septic group compared to both the ill group (*p* = 0.006) and orthopedic control groups (*p* < 0.001). Body temperature was also significantly higher in the septic group compared to the ill control (+0.8 °C; 95% CI 0.02–1.5 °C; *p* = 0.044) and the orthopedic control groups (+1.0 °C; 95% CI 0.4–1.7 °C; *p* = 0.001). 

## 4. Discussion

This retrospective study compares resting serum bile acid concentrations in a group of dogs diagnosed with sepsis and a population of non-septic dogs divided in two controls groups: one of ill dogs, diagnosed with a non-hepatobiliary/pancreatic systemic disease, and another control group of dogs that underwent an elective orthopedic procedure, considered otherwise healthy. 

When evaluating resting SBAC concentrations between the septic and both control groups considered together, resting SBAC were significantly higher in septic dogs, which is in agreement with reports from the human literature, in which higher serum bile acid concentrations have been reported in patients with sepsis, and are also considered an early predictor of short-term survival [10,11,15,16,17]. Serum bile acids are used as biomarkers for changes in the enterohepatic circulation secondary to intestinal, hepatic, or infectious disorders [26] and as cholestasis is a common complication of sepsis, increased serum levels of bile acids are deemed to be predictive of sepsis-associated mortality [10,17]. 

When comparing the septic group to both control groups individually, differences were identified between the septic and the ill control group, with dogs in the septic group displaying higher resting SBAC. However, the concentrations were not significantly different between the septic group and the orthopedic control, which was an unexpected finding. When considering orthopedic group, albeit not reaching statistically significance, dogs included in this group had a higher median age. Furthermore, there was a significantly higher proportion of female dogs in the orthopedic group compared to the other groups. 

A study in the human literature evaluating the effect of ageing on conjugated and unconjugated serum bile acid levels in healthy subjects has not found significant differences in fasting serum bile acid concentrations between a population of elderly (older than 60 years) and younger healthy patients [27]. The same study concluded that that postprandial conjugated bile acid concentrations were higher in younger subjects [27]. Moreover, further studies exploring age-related changes in the lipid metabolism, have suggested an inverse correlation between bile acid synthesis and age [28,29]. 

When considering the effect of sex in bile acid concentrations, a study in the human literature investigating this influence has concluded that fasting plasma concentrations of several bile acids were affected by sex, with men having a higher mean concentration of total bile acids when compared to women [30]. This finding has been supported by further studies [31,32,33]. 

There is scarce information in the veterinary literature regarding the effect of age and sex in bile acid concentrations. A study assessing the effects of age, breed, sex and endocrine disease in plasma cholesterol and lipoprotein concentrations has concluded that these were unaffected by neither age or sex [34]. Although plasma bile acids were not considered in the aforementioned study, bile acids play a key role in cholesterol and lipid homeostasis [35], thus it is plausible that similar conclusions could be inferred with bile acid concentrations. Moreover, no correlation was found between resting SBAC and age in the present study. It is therefore unlikely that the higher median age for dogs in the orthopedic group could have influenced the resting serum bile acid results in this group. The reason why female dogs exhibited higher concentrations of resting SBAC remains unclear, as it is not supported by the human literature. One possibility is that there might be pathophysiological differences in the effects of sex on serum bile acids in veterinary medicine. Alternatively, this might represent a coincidental finding. 

There are several other possible explanations for the lack of significant differences in resting serum bile acids between dogs in the septic and the orthopedic control group. Firstly, and the most likely reason, is that the results in the latter group have been influenced by outliers due to the small sample size, as although no statistically significant differences were found, median resting bile acid concentrations were numerically higher in the septic group. Secondly, another possibility that cannot be excluded due to the retrospective nature of the study, is that although patients presenting to this referral center are asked to be starved, it is not possible to confirm whether all the patients in the orthopedic control group had been starved prior to presentation, which could have resulted in the higher resting bile acids documented, in the absence of liver disease. This hypothesis is unlikely to apply to the dogs in the septic group, as most of these patients presented as emergencies, therefore unwell and hyporexic, and serum bile acid concentrations in this group are more likely to represent a starved sample. Thirdly, even though dogs included in the orthopedic group were deemed healthy, they could have had an undiagnosed underlying condition leading to cholestasis. Interestingly, all dogs in the orthopedic group had received a non-steroidal anti-inflammatory drug (NSAID) for at least two weeks prior to presentation. It has been proposed that changes in the microbiota due to NSAIDs administration can alter the composition of bile, with increased cytotoxicity of bile salts due to suspected micellar incorporation of NSAIDs [36]. A study assessing the effects of firocoxib in geriatric dogs over a period of 90 days found statistically significant differences in bile acids between day 0 and day 90 [37], and it was suggested in the same study that this change in bile composition secondary to NSAIDs could affect bile flow and cause cholestasis [37]. Although dogs had not been administered NSAIDs for the same length of time as the one considered in the aforementioned study, it can be hypothesized that a similar mechanism could have contributed for higher bile acid results in the orthopedic control group. 

In the present study, dogs in the septic group had significantly higher bilirubin and lower albumin concentrations compared to the ill and the orthopedic control groups, which is in agreement with other studies in the literature regarding patients with sepsis [4,21,23,24,38]. Moreover, similarly to the findings documented in the human literature, in the study evaluating circulating bile acids in critically ill patients [10], a positive correlation was also identified between resting SBAC and serum bilirubin. 

No significant differences were found in serum cholesterol concentrations between groups, which is an unexpected finding. A retrospective study evaluating plasma cholesterol concentrations in septic dogs, concluded that higher plasma concentrations of cholesterol correlated with a higher probability of survival, suggesting that plasma cholesterol concentration could provide prognostic information in dogs with sepsis [19]. This conclusion is supported by studies in the human medicine literature, in which hypocholesterolemia in septic patients is associated with a poor prognosis [39,40,41]. The small sample size in the present study could have precluded detection of differences in cholesterol concentrations. Moreover, dogs were admitted at different stages of disease progression, and therefore the impact of sepsis in cholesterol measurements may not have been significant. 

Septic dogs had higher heart rates, temperature and neutrophil counts compared to control groups, which is also in agreement with a previous study [23].

Overall survival in this study was 46% which is in accordance with the published literature regarding canine sepsis [4,19,20,24,42,43]. 

There are several limitations to this study, most of which secondary to its retrospective nature. A large number of cases with incomplete data were excluded from statistical analysis, resulting in a small sample size. This limited the ability to more accurately compare variables and detect small differences between groups. A power calculation could have been performed prior to the study to ascertain how many cases were needed to answer the study hypothesis. Nonetheless, the number of cases included in the study would always be limited by the number of patients that presented at the referral center during the time period considered, as this is a single center retrospective study. Therefore, it is possible that the study is underpowered which increases the likelihood of type II errors occurring.

Serum bile acid concentrations were only evaluated at a single time point, on admission, which constitutes another limitation of the study. A study assessing resting and postprandial serum bile acid concentrations in dogs with liver disease documented an overlap in these parameters in different hepatic conditions in dogs, concluding that single bile acid measurements are of limited utility [44]. Moreover, the same study documented resting SBAC of <10 µmol/L and >90 µmol/L in dogs with different categories of liver disease confirmed by histopathology [44]. The use of both resting and postprandial bile acids provides the most reliable information [35], however measurement of postprandial SBAC is a more sensitive marker than resting SBAC for the diagnosis of liver disease [45]. Although postprandial bile acids could have been helpful in lending support for a diagnosis of cholestasis, this study has only assessed the resting bile acids since this parameter is part of routine biochemistry analysis and was not specifically requested for the dogs included in the study. Moreover, performing a bile acid stimulation test in septic dogs could be challenging, as most of these patients present hyporexic and placement of feeding tubes may not be possible in all cases. Additionally, for the control groups, postprandial sampling may not be possible if patients require surgery and should therefore be starved, or ethical if that test is not clinically indicated. Furthermore, in the aforementioned study evaluating resting and postprandial serum bile acid concentrations in dogs with liver disease, in the five dogs with a histopathological diagnosis of cholangitis (which leads to intrahepatic cholestasis) and that had also a bile acid stimulation test performed, none had a marked increase in the postprandial sample compared to the resting sample, with three dogs having a lower postprandial SBAC compared to resting concentrations [44]. Therefore, even postprandial serum bile acid concentrations should be interpreted cautiously in patients with cholestasis. 

It should also be emphasized that any extrahepatic disease can result in a non-specific reactive hepatitis with subsequent cholestasis and differentiating the latter from sepsis-induced cholestasis is challenging. Therefore, although statistically significant differences were identified in resting SBAC between the septic and the non-septic groups considered together, causation cannot be proven. 

Finally, there are several diagnostic criteria in the literature to define sepsis and SIRS, and possibly the use of different criteria could have resulted in the inclusion of more cases and different results. 

## 5. Conclusions

In summary, resting serum bile acid concentrations were significantly higher in septic dogs compared to a population of ill, non-septic dogs, despite not differing between the septic and an apparently healthy control group. Therefore, these results should be interpreted cautiously due to the small numbers of cases included in the study. Furthermore, further prospective studies assessing the effect of the use of NSAIDs in bile composition could be considered, bearing in mind our findings. 

## Figures and Tables

**Table 1:** Summary of results for septic, ill control, and orthopedic control groups. Values presented as mean (±SD) for normally distributed continuous variables and median (IQR; range) for non-normally distributed continuous variables.

Variable	Septic Group	Ill Control	Orthopedic Control
Heart rate (bpm)	127 (±28.6)	105 (±18.7)	101 (±18)
Temperature (°C)	39 (±1.4)	38.2 (±0.4)	38 (±0.6)
Glucose (mg/dL)	93.6 (±16.2)	102.6 (±10.8)	108 (±12.6)
Neutrophil count (×103/µL)	9.5 (3.14–21.74; 0.06–126.8)	7.38 (5.16–8.45; 3.5–21.2)	4.62 (3.16–6.05; 2.3–12.2)
Albumin (g/L)	20 (16–25; 9–35)	31 (28–33; 24–37)	34 (31–36; 26–39)
ALP (U/L)	165 (113–288; 17–2037)	50 (34–74; 28–1103)	58 (29–137; 14–843)
Bilirubin (mg/dL)	0.26 (0.18–0.88; 0.06–2.81)	0.18 (0.12–0.23; 0–0.29)	0.12 (0.06–0.18; 0–0.23)
Serum bile acids (µmol/L)	17.5 (7.5–26.9; 3–75.2)	6.9 (4.7–14.4; 2–47)	12 (9–14; 3–32)

## Data Availability

The data presented in this study are available on request from the corresponding author.
